# Book Reviews

**Published:** 1818-02

**Authors:** 


### CRITICAL ANALYSIS

### *OF RECENT PUBLICATIONS,*

IN THE

### DIFFERENT BRANCHES OF PHYSIC, SURGERY, AND MEDICAL PHILOSOPHY.

---

*A Narrative of the Case of Miss Margaret M'Avoy; with an Account of some Optical Experiments connected with it.* By THOMAS RENWICK, M.D. Physician to the Liverpool Infirmary. 4to. pp. 126. Baldwin and Co. London.

*Hints to Credulity; or, an Examination of the Pretensions of Miss M. M'Avoy, occasioned by Dr. Renwick's Narrative of her Case.* By JOSEPH SANDARS. 8vo. pp. 69. Liverpool: printed for Longman and Co. London.

——"Ingenuos didicisse fideliter artes

Emollit mores, nec sinit esse feros."

SUCH are the fond expectations we entertain from the cultivation of the mind, from the *belles lettres,* the *artes humaniores,* the το πρεπος, the καλοκαγαθια, and, above all, from the sure, though slow, progress of Christianity. Alas! how are we deceived! Yet we should not entirely forget that more warmth has been evinced on subjects much less important than the present. We may, however, be allowed to regret that the whole question has not been treated with that philosophic calmness, that conciliating urbanity, which must ultimately have brought conviction to every mind, without injury to the feelings of any.

In considering the publications before us, we shall confine ourselves as much as possible to extracts. We have had the happiness of knowing, and of respecting, many of the characters who are advocates on each side; but none of those whose severity, to use the most gentle terms, we are forced to condemn. Not that we have any doubt of the conviction of either party to all they assert; but, wherever passion mingles in controversy, the most violent are the most likely to be led astray.

Miss M'Avoy, it seems, had been dull in her hearing. After the long continuance of a severe illness, attended with convulsions, a quantity of fluid seemed to pass from the head to the fauces, and thence to her stomach, from which some was thrown. About seven ounces, before it reached beyond the nose and fauces, were caught in a bason, and, being analyzed by Dr. Bostock, evinced nothing very particular. From this time the convulsions ceased in great measure, but part of the body and extremities appeared paralytic.

Some time afterwards, though the patient had not recovered her sight, she was enabled to amuse herself with sewing, knitting, and other female occupations, in which by habit she gradually improved. Her mother wished her to fix her eyes as if she were looking intently at her work.

"Miss M'Avoy (we are told) endeavoured to follow this advice, and applied with so much perseverance, that she succeeded not only in this point, but sewed much better than ever she had done before her deprivation of sight. Mr. Thomas and I particularly examined her eyes, exposed to the light of the candle, as near to the eye as possible without burning her, but without observing the slightest contraction or dilatation of the pupil, or the least sensibility in the eye. I have frequently, since this period, thrown my hand suddenly towards her face; have pretended to dash a pointed penknife at the eye; and have often applied the point of the finger, in a quiet and steady manner, as near as possible to the pupil, without observing the slightest sensation in the eye. Mr. Thomas assured me he has more than once put his finger upon the cornea itself, which then appeared insensible; but, when he touched the eye-lid or eye-lash, she was instantly sensible of it. I have sat for a considerable time attending to her sewing, but apparently indifferent about it; and, during these visits, I have examined her every action as minutely as possible, and I have been satisfied she could not see. In threading her needle, it sometimes required only one effort, but often three or four, before she was successful. It was curious to observe her when, by any accident, the point of the thread was bent: she would try to thread the needle three or four times, and, if she did not succeed, would put it into her mouth, and bite off a part, or take her finger and feel whether the thread were straight or not. More than once I have given her the needle with the point upwards, when she had accidentally dropped it: she attempted, but, finding after a few trials that she could not thread it, she would put her finger upon the needle, and, feeling the point, would turn and thread it: once, also, I broke the eye of a needle in drawing the thread hastily out, but was not aware of the circumstance until Miss M'Avoy had made several attempts to thread it; but, failing, she felt the eye with her finger, and told me it was broken, which, upon examination, I found to be the case. The circumstance of Miss M'Avoy's knitting and sewing did not appear to be so very extraordinary, as there are many blind persons who are capable of doing the same.*

* It may be mentioned, that the blind people in the Asylum generally make use of the mouth and tongue in threading their needles. In the Blind Asylum of Liverpool, blind persons are

In a subsequent page, Dr. Renwick proceeds to give an account of the manner in which this lady discovered her power of seeing with her fingers.

"It was very early in September that her stepfather, Mr. Hughes, was reading a few pages in a small book belonging to one of his children, in which a history was given of the life of St. Thomas à Becket, not very favourable to his general character. He mentioned it to his wife and daughter, and said he recollected having read once a very different account in another book. Miss M'Avoy told him that she had, before she was taken ill, seen an account of his life in a book entitled the Lives of the Saints, and, if she had the book, could point out the place where it was. The book was put into her hands, and, in turning over the leaves, she pointed out the place, and read a few words. In a jocular manner, Mrs. Hughes asked her if she could feel the letters with her fingers. She said she had felt the words she had read, and would try, if her father would give her a book. A number of a folio Bible, of tolerably large print, was given, and she read several verses, to the great astonishment of her father and mother. Upon hearing this account, I was induced to visit her again with Mr. Thomas, and took considerable pains in examining the eyes; but we found little or no alteration in their general appearance, except that the pupil was not quite so much dilated as before; but the light of a candle appeared to have no influence upon it. We found her father's account very accurate, and that she really could read by the application of the fingers to the letters with considerable fluency. As it was probable any other person, who had not the same opportunity of judging with Mr. Thomas and me, might think it possible she could see, I thought it right to bind something over the eyes, and I made use of a Manchester cotton shawl, which went twice round the head, crossed at the eyes, and was tied at the back of the head as firmly as she could bear it. I placed in her hand a number of the Bible above-mentioned, and she read very correctly one verse of a chapter in Genesis. I then requested to have another book, which happened to be a volume of the

---

not only taught to sew, knit, and spin, but become expert in the making of baskets of various descriptions, plain and coloured, twine, ropes, bears, carpets, rugs, slippers of list and worsted: a few make considerable progress in music, and often become teachers in their turn to others. The most of them, in a few years, are enabled to follow the different trades they have been taught, and to support themselves with credit and independence at their own homes. This institution owes its origin to the Rev. Henry Dannett; and, after his secession from Liverpool, its prosperity was increased by the great attention and unwearied zeal of the late Pudsey Dawson, esq. supported by the humane subscriptions of the inhabitants, and by strangers, who make it a principal object of their attention in their visits to Liverpool."

Annals of the Church. I opened it, and she read to me several lines, with the alteration in a proper name of only one letter, which, upon being desired to read over again, she corrected. I then turned to a few lines of errata, and she read them correctly, only reading the letter I. as an I and a dot. The mode she follows is to place her fingers upon the book, and, when she feels the letters, to proceed from the beginning to the extremity of the word, and back again until she names it, and so on to the next word. She often makes use of the fingers of both hands, particularly the fore-fingers, and, when they are in good order, she will read from twenty-five to thirty words in half a minute.

"On the following day I mentioned the circumstance to a friend, who was anxious to see a phenomenon of this kind, and he met me in St. Paul's-square. Miss M'Avoy again read over to us a verse in the Bible, a few lines in the Annals of the Church, and the title-page, mottoes, and several lines, in a 12mo. edition of Grahame's Sabbath. I placed her fingers upon a blank leaf, and desired her to read. The attempt was made, but she said she could not feel any letters. Her fingers were then placed upon another leaf, which she declared was also blank. I then desired her to feel the upper part of the leaf. She did so, and said she felt something, but it was so confused she could not make out what it was. The fact was, a lady's name had been written in the book, and, when I took it from my library, I scratched the name out with a pen, so that it was not distinguishable to the eye. My friend brought with him a French assignat, of which it was more than probable Miss M'Avoy before did not even know the name. She read it over, and mentioned the lines upon it as distinct from the plain paper, and the colour of them, which was black: she also decyphered the address and post-mark of a letter received by that morning's post; she named the colours of the coats, waistcoats, and pantaloons, of the gentlemen present with accuracy. She gave a correct account of a few cards, one or two of which were put under a small table placed before her. My friend Mr. Egerton Smith, who accompanied me, visited her afterwards several times by himself."

This last-mentioned gentleman took with him Mr. Nichol, a scientific character, at that time delivering a course of philosophical lectures; and the events of this interview were so remarkable as to find a portion of the Liverpool Mercury for their relation.

After this, some suspicious events occurred at Wavertree, which are accounted for by the agitation of Miss M'Avoy's mind. Even the author doubted: but, from subsequent experiments, was again convinced of her powers.

"Her character for truth (it is urged) and integrity is attested by her confessor, the Rev. Edward Glover, which, in a moral point of view, convinces him she does not deceive. The evidence of Mr. Glover, Mr. Thomas, and myself, will have some weight upon the public mind; but I cannot expect it will satisfy every individual. Every facility has been given to those who had the curiosity to visit Miss M'Avoy, of which numerous parties have availed themselves; and they are convinced, at least, if she were not blind, that she possessed the power attributed to her, when blind-folded. Several, who imagined themselves to be more philosophically gifted, declared, although the eyes were allowed to be perfectly covered, this faculty must be deceptive. Other persons asserted her eyes must be more peculiarly acute, and that she could see with the smallest quantity of light; a very few declared she must see sideways, downward, or even backward; and one individual amused the party he accompanied, by saying she could see through the nose, which he had known to be the case in another instance: in fine, the generality became proselytes to the evidence of their senses, but a few were found who doubted that evidence. Dr. Trail and Dr. Formby visited her separately after the Wavertree examination, but neither of them was satisfied; although, when the latter attended, other persons were present to whom she named several colours of silks, cloths, wafers, &c. but she never could name a colour to him, unless it were one which he held above her head. In prosecuting the narration of Miss M'Avoy's case, an account of the examination at which Dr. Trail was present will be given. Mr. Kent visited her with me, and did not hesitate to alter the opinion he had formed at Wavertree. Dr. Jardine also saw her again, and, to satisfy himself still more, he repeated his visits very often, and is as perfectly convinced as man can be, who has paid particular attention to the subject, that she possesses the power of reading with the fingers, distinguishing colours, &c. On the contrary, several individuals of respectability, who have seen her, and been satisfied she possessed the power, have altered their opinion, which may have arisen from trifling circumstances every person is eager to catch at, exciting suspicion of deception."

"On the 31st of October, I have been informed by a lady, that Miss M'Avoy visited Mr. Charles Clements, in Queen-street. There was little fire in the room, and it was so dark that candles were brought in, but were afterwards ordered away, from the wish they had to hear Miss M'Avoy read, &c. A book just published upon Brewing was put into her hands, and she read a few lines correctly, but, in attempting to read the word vat, she only made out a v and t. Mr. Clements was induced to take the book, and he had some difficulty, from the small quantity of fire in the grate, to distinguish the words: he, however, found it out to be the letter a, turned upside down. She told also the colours of different substances given to her very accurately, in a light by which no other person could distinguish them, and when the persons were standing between her and the fire.

"The effects of the Wavertree expedition, and the agitation she suffered, soon became visible, in a return of the pain in the head and right side, which obliged her to have recourse again to the mercurial friction, which relieved the pain in the side. The pain in the head was again attended by the beating sensation or throbbing, with loss of appetite and giddiness."

A severe illness followed, which so debilitated the patient that for a time she lost the power altogether: as she recovered her health, the power returned, and the number of visitors was so great that her physician advised her retirement into the country, to avoid the consequences of over-fatigue.

"On the 19th of April, Dr. Jardine requested that he might visit Miss M'Avoy, before she went into the country; and an appointment was made at eight o'clock in the evening. I called upon Dr. Jardine, and found at his house Messrs. Bickersteth, senior and junior. We proceeded to St. Paul's-square. Mr. Thomas was also present. The mode of covering the eyes this evening was one I had tried, for the first time, a few days before, and consisted of a piece of gold-beater's skin, sufficiently large to cover the eye, extended, and sewed upon two pieces of velvet, which adhered to each other over the nose.

"The edge of the velvet could be turned up, so that, when the gold-beater's skin was wetted and applied to the eyes, and then allowed to dry, it appeared closely adhering to the skin. This mode of covering was pleasanter to Miss M'Avoy than any other I had before used; and it was a cooler application than either the gogglers or the Manchester shawl. She named the colour of every piece of silk that was given to her, except one, and that she told afterwards. A piece of green silk was inclosed between two pieces of glass, and the edges of the glass were sealed with sealing-wax: upon feeling the outside of the glass, she said the colour was green; a piece of red silk was inclosed in a similar manner, which she named correctly. She told the hour and minutes in two watches, which differed in time from each other. When placed in a situation of complete darkness, three cards were given to her: the two first were clubs, and the last hearts; they were all black. A green and black plaid was given into her hands also, when the room was quite dark; but it was black to her feeling. Upon Dr. Jardine opening the door, a feeble light was thrown upon a part of the plaid, and she then declared it to be green and black.

"During some of the former examinations, Dr. Jardine made use of an egg to cover the eyes, prepared by boiling until it became quite hard; it was then divided longitudinally, and the yolk taken out. Dough was also tried, and, if made of a proper consistence, and kneaded well, answered every purpose. A ribband applied over the eyes, so as to leave the upper and lower parts of the egg and dough visible, made the experiment more satisfactory.

"It was about this period that Miss M'Avoy endeavoured to amuse herself in making small baskets of coloured paper: it was curious to see her passing the paper through the interstices of the basket-work. She was often foiled by the point of the paper being turned inward or outward. If she found she did not succeed after two or three attempts, she used her fingers to straighten it, and then pushed it through. She sometimes used a pin or needle to raise the paper under which the point should pass."

Another illness followed. After convalescence,

"The eyes were covered with the black velvet and gold-beater's skin, with a silk handkerchief tied over the whole. She read several lines which Dr. Brandreth wrote, with tolerable precision; and, when she mistook a letter, it was more like what she named than what Dr. Brandreth intended, as it was written in great haste She told some letters upon a snuff-box, which could not be read easily without a glass; and, with a magnifying glass, she read all the words but the termination of the last. She traced with her fingers the landscape, which consisted, amongst other objects, of two cocks fighting; she said they were like two peacocks: the tails of the cocks were very full, and we did not think her remark much out of the way. The lines at the bottom were—"Better stuff never trod a midden." She told the time of the day, and several colours. Upon taking off the handkerchief, one of the pieces of gold-beater's skin was loosened from the eye, but they appeared still to be sufficiently covered by the handkerchief."

A few days after, Dr. Renwick was particularly anxious that the gentlemen who were rendered sceptical by the events of Wavertree, should repeat their experiments at Liverpool. The attempt was made, but it proved as unsuccessful as before. On the following day, her eyes being covered with sticking-plaster and black silk, and a silk handkerchief tied over the whole, crossed at her eyes and pinned above her ears, she distinguished colours, read, and told the hour of the day by two different watches.

On a subsequent occasion, she perceived the sun through a pane of glass, and also its reflected image; and was not dazzled with its light, but found it pleasant. Several articles held over head she perceived in her plain glass; and, with her fingers on the window, described things in the street; did the same with her back to the window, and her hands behind her.

On another occasion, a large pasteboard was cut

"So as to admit the nose, and to press upon the cheek, and formed a sort of grenadier's cap, rising above the head. Cotton-wool was sewed upon the edges, where it touched the nose and cheek, both inside and out. Tapes were attached to it in two or three different places, which were tied round the back of the head, and a small piece of tape closed it still more over the nose. Applied to my face, and to that of several other gentlemen, we could not see; but, if it were put upon the nose of any gentleman which was more prominent, it did not fit well, and a person thus constructed might see and describe colours. The same remark might be made upon the gogglers; but, when the cross string over the nose is tied firmly, it is scarcely possible for any one to see so as to distinguish any object. At any rate, Miss M'Avoy's eyes are completely covered with it, as, by looking from above down to the nose, I could see no object. The cotton, wool, silk, and cloth, were given to her, but she did not distinguish any of them. Thursday was very hot and gloomy. The hands and fingers were warm, but there was a clammy moisture upon them, which appears to take away the feeling as much as when they are cold."

Covered in this way, "she was desired to feel upon the glass, and to describe the object passing. She answered accurately, that the lady's gown was white; that she had a blue spencer on, and a green umbrella in her hands. She distinguished the colour of a supposed cairngorum stone: the colour she named, but she said it was not a stone, but glass; and so it proved to be by a file."*

Those who feel interested in the question will refer to the work; others will, perhaps, accuse us of having already given too long and too minute an account. At length, a large meeting took place, in which Miss M'Avoy did not succeed so well as usual; which Dr. R. imputes to her being made acquainted with a letter received by Mr. Hughes, her stepfather, and ill-advisedly communicated to her. The publication of this letter gave rise to Mr. Sandars' Tract. The narrative concludes thus:—

"Since this narrative went to the press, several parties have visited Miss M'Avoy, and have declared their conviction of her being blindfolded, and of being able to distinguish colours, read, tell the time of the day, &c. Amongst the number who have seen her are, the bishop of Chester, the vicar of Bolton, Lord Lilford, Mr. Ireland Blackburne, and Mr. Blundell, of Ince.

"The Rev. Jonathan Brooks, Mr. Thomas Cropper, Mr. John Cropper, Mr. Thomas Corrie, Mr. Shuttleworth, Mr. W. Ingram, and a very large party of ladies, visited Miss M'Avoy on the 13th of October. Miss M'Avoy's eyes, particularly the left, were so sore, that the gold-beater's skin upon black velvet, with a piece of plain silk placed in the centre, was only applied to each eye. The covering was not as satisfactory as I could have wished, nor do I think it would convince any of the party that Miss M'Avoy was blindfolded; of course, all the experiments made on this day would be nugatory. Some of the party thought the gogglers more effectual, and she readily assented to have them tied on. She ap-

* "These stones have since been shewn at Mr. Harper's shop, in Lord-street, and they say they are cairngorum stones."

peared, however, not to be able to tell a single colour, having been very much exhausted during the first trials."

Having finished the history, some remarks follow on the case. Till the main point is proved, these will be of little consequence. Dr. Renwick seems to consider, and we think with much propriety, that the fluid which escaped from the head was the contents of an hydatid. Some observations follow on his own conviction of the young lady's blindness, and the doubts of others. The following letter from Mr. Brandreth, who ranks very high as a consulting surgeon and occulist, we conceive worth copying.

"My dear Sir,—In compliance with your request, I have repeatedly examined Miss M'Avoy's eyes, with all the attention so important a case requires. It is the firm conviction of my mind that she is really blind.—With respect to the iris, I have no doubt that, at some period or other, it has been much inflamed, and that adhesion of some of the fibres with each other has been the consequence. I judge this to be the case, from the seemingly capricious manner, if I may so speak, in which it contracts and dilates, although in neither case to any great extent. I have more than once used a strong solution of belladonna to the eye and surrounding parts, without producing any sensible dilatation. The first time I used belladonna, it seemed to enlarge the pupil of the right eye to a certain extent, but by no means in a similar way to what occurs in a healthy state of the iris. I have sometimes seen it more relaxed in a bright light than in a weak one. I have thrown the light of a candle on the centre of the cornea, through a powerful double convex lens, without its contracting in the slightest degree.

"I am, dear Sir, yours truly,

"J. Brandreth."

---

A correspondence between Mr. Hughes, the stepfather of Miss M'Avoy, and Mr. Sandars, produced the pamphlet second in order. From this we shall offer a few extracts.

The first of the following letters is without a name. The second from a gentleman well known at the Coffee-house, as Lloyd's is commercially called in London, not only as a good man, to use the same local phrase, but as a man of considerable abilities and information, and arrived at an age when he is likely to consider every question with due caution.

"What then would Miss M'Avoy wish the world to believe? She declares the breath of her nostrils to be necessary to her performances; she proves that it is not: she asserts that light is indispensable; it is shown that she can at least read without it: and, if either touch or the application of breath be requisite, how can she distinguish persons walking in the street, by turning her back and fingers to the window?"

"The gogglers were applied. A book, which a gentleman present happened to have in his pocket, was given her: after passing her fingers repeatedly over a particular line, she placed the book upon her knee, and, covering her right hand with her left, she read as follows:

'I will not name them, replied Zelia.'

The line, as printed, ran thus—

'I will not name them, was Zelia's answer.'

"A piece of crimson and white paper was put into her hand, with the coloured side down: after feeling at it some time, she decided that it was black and white. On afterwards holding the paper up to the light, the crimson colour had very much the appearance of being black.

"The same piece of paper, with the coloured side up, was again given to her, (a sheet of writing-paper having been previously interposed between her face and her hands): she said, she could not tell what colour it was. On her saying this, the sheet of paper was withdrawn, when she immediately told the colours correctly.

"A letter was then given her, (the sheet of paper being interposed, as in the last experiment,) and she was requested to name the colour of the wax with which it was sealed, and likewise what were the letters upon the seal. She said, the seal was black, but she could not distinguish what the letters were. The seal was red, and the letters upon it were sufficiently large to have enabled a person with any delicacy of touch to have told what they were.

"It is presumed that this gentleman's testimony (whose name I am not authorised to communicate publicly) proves that she has not a very exquisite sense of touch. From the error committed in reading the line, a very strong presumption arises that she got it by rote, previously to covering her hand, and that her memory betrayed her: such enlightened fingers never could mistake whole words. The next letter will likewise prove that her sense of touch is not very refined; and it cannot fail to strike the reader how easily she distinguished the colour of the gentleman's hair, when placed in the line of vision. What is the inference?

"'Dear Sir,—In compliance with your request, I will endeavour to recollect some of the circumstances that occured at my short visit to Miss M'Avoy. I was introduced by a friend, and found assembled a very numerous and highly respectable company of ladies and gentlemen; it was not at the house of her stepfather, but, as I understood, the adjoining house, the parlour of which was more commodious. I kept no memorandum, but, on reference to Dr. Renwick's publication, I observe, by the names of part of the company, that it must have been on the 23d of September.

"'After blindfolding the young lady in the usual manner, a watch was given into her hand, and she told the time exactly, that it wanted six minutes and a half to three. The routine of experiments were then gone through, of coloured cards, gowns, shawls, silks in a phial, and also the mirror. This she held in her hand, and a gentleman looked into it over her shoulder. She could not make out any thing: I remarked to him, that, if he would lean further over, so as to see the lady's face in the glass, she would more readily receive the impression; he did so, and she then described his countenance and the colour of his hair. She was soon after desired to feel the hair of Mr. William Earle, and tell the colour. She either said positively, It is red, or interrogatively, Is it not red? This gentleman's hair is very grey, and he wears powder. She was next desired to describe the colour of the hair of Mr. Earle, who sat nearer to her, and more forward: she felt it, but said she could not tell.

"The room being heated by the crowd of company, she was advised to retire for awhile. In her absence, I suggested to Mr. Earle, that, if the young lady would permit him to lay his head in her lap, I was confident she would discover the colour of his hair. Immediately on her return, he did so; and, without hesitation, she said it was white. The elder Mr. Earle does not wear powder, but his hair is also very grey. Some few other experiments were made, when this gentleman said, he had heard that Miss M'Avoy could discover by the feel what was written on the inside of a hat, and he presented to her the concavity of his own hat sideways: she put her hand into it, and said she felt nothing. On this a card was given to her, coloured on one side a bright red, and on the other blue: she felt it a long time, and at last she could not distinguish the colour. Here some one remarked that the *faculty* was gone, or going.

"'I must confess, that, from what I had witnessed, it appeared to me that this intermitting of what is called the young lady's faculty, together with the alleged necessity of a free communication between her breath and the object to be distinguished by the touch, are occasionally extremely convenient.

"'Yours, dear Sir, very truly,

"'*4th November*, 1817. J. T. Koster.'

"'*To Mr. Sandars.*'

"The next communication is from the pen of a medical gentleman, whose accuracy of observation is well known to his friends.

"'*Liverpool*, *Oct.* 31, 1817.

"'Dear Sir,—In compliance with your request, I purpose stating, as accurately as I can, my observations during a visit I made to Miss M'Avoy, I think, on the 2nd of September last. I shall confine myself solely to facts.

"'Dr. Jardine kindly obtained permission for me to see Miss M'Avoy, and introduced me to her.

"'As I arrived some time before Miss M'Avoy made her appearance, I had an opportunity of examining into the means employed for covering her eyes, when experiments are made. I

was shewn the pasteboard screen described in Dr. Renwick's "Narrative," and the mask with the goggles, of which the Doctor has given a sketch in the frontispiece to his publication. Wishing to satisfy myself that the gogglers were an effectual blind, I had them tried on myself. Dr. Jardine tied the strings tighter, I believe, than Miss M'Avoy could have borne. On looking down my nose, I found I could distinguish objects immediately below me, without difficulty. I saw my watch-chain, for instance. I was glad to get rid of the mask; it made me extremely hot, and occasioned an uneasiness in my eyes, which continued several hours.

" 'When Miss M'Avoy entered, her hands were hot, and the power had left her. I asked permission to examine her eyes, which she granted with great good humour. They appeared somewhat dim, and placed deep in the head. On exposure to a strong light, the pupils contracted as much as those in healthy eyes, under similar circumstances, but I think rather more slowly. I took an opportunity of darting my finger rapidly towards the eye; it did not blink.

" 'Miss M'Avoy, after cooling her hands with a wet sponge, said she would try whether the power was returned, before her eyes were covered. Accordingly, some bits of coloured silk were presented to her one by one. I was standing close to her, and looked intently at the eyes, to watch their motions. The three colours first given her were blue, scarlet, and pink: she named them rightly, after having felt the bits of silk for a few seconds; but I observed the eyes were previously directed to the objects, with a rapid and instantaneous glance. The next bit of silk was of a drab or fawn colour; she did not tell its colour, nor were the eyes directed to it.

" 'The pasteboard screen, cut to fit the face, and having its edges covered with cotton-wool, was then applied. On looking under it, rays of light were perceived between it and the nose. These I effectually excluded, by gently insinuating a little cotton into the interstices that admitted the light, so that it was impossible she could see any object placed immediately under the pasteboard, even if she were not blind. Unfortunately, the power now left her, and she failed in every attempt. In answer to a question I put to Miss M'Avoy, she said she preferred the gogglers to the pasteboard.

" 'Miss M'Avoy now retired, and was absent for a considerable time. On her return she appeared cheerful, and said she believed she should now succeed. The gogglers were put on. She distinguished colours, told the time of the day by a watch, read a few words from a book, a written note, and an address card, in her usual manner. During these wonderful performances, one of the company interposed a piece of printed calico between her face and the object; she immediately perceived it, and pushed it away in rather an angry manner. On the gogglers being taken off, a gentleman of high rank in the navy closed the eyelids with his fingers; the power instantly left her, though a few seconds before she seemed in full possession of it. I regret I could not prolong my visit, and witness a few more performances of this extraordinary young lady.

" 'I have now given you a full, and somewhat desultory, account of what I saw on my visit to Miss M'Avoy, and have stated impartially every circumstance that might appear either to confirm or invalidate the idea of her being blind. I shall make no comment; but, comforting myself with the conviction that 'truth is mighty, and will prevail,' I subscribe myself your sincere friend,

" 'C. Worthington.' "

Such is the substance of this controversy, divested of those personalities which probably are the offspring of passions necessary to stimulate an individual before he engages heartily in opposition of any kind. It is not our intention to condemn such feelings: they are a part of our nature; but we wish the expression of them should be restrained, and are certain the success of the combatants would be secure in proportion as their hostility is concealed, or at least mitigated. For ourselves, we have no object but to fulfil our duty in preserving a faithful record of whatever is connected with medicine.

---

*A Practical Inquiry into the Causes of the frequent Failure of the Operations of Depression, and of the Extraction of the Cataract, as usually performed; with the Description of a Series of new and improved Operations, by the Practice of which most of these Causes of Failure may be avoided. Illustrated by Tables of the comparative Success of the new and old Modes of Practice.* By Sir William Adams, Member of the Royal College of Surgeons in London; Occulist to his Royal Highness the Prince Regent, and others of the Royal Family; Corresponding Member of several Learned Societies; and late Surgeon to the West-of-England Infirmary for curing Diseases of the Eye, instituted at Exeter. 8vo. pp. 413. Baldwin, Craddock, and Co. London.

*A Letter to the Right Honourable and Honourable the Directors of Greenwich Hospital, containing an Exposure of the Measures resorted to by the Medical Officers of the London Eye Infirmary, for the purpose of retarding the Adoption and Execution of Plans for the Extermination of the Egyption Ophthalmia from the Army, and from the Kingdom, submitted for the Approval of Government.* By Sir Wm. Adams. 8vo. pp. 152. Baldwin and Co. London.

The first of these works is introduced by a dedication to the Right Hon. and Hon. the Directors of Greenwich Hospital, in which the author reminds them of his success in curing the Egyptian ophthalmia by an improved plan of his own: of this we shall have occasion to speak hereafter.

In the Preface, we are told that all the operations described in the present work have been detailed in a former Treatise of the author, excepting his new method of extraction. Here Sir William thinks it necessary to apologise for changing his practice, lest he should be suspected of "vacillation of opinion." We should rather have expected an apology for swelling the present book with the contents of the former, than for improving his art, and acquainting the public with such improvement. Sir William proceeds,—"The works that have heretofore appeared upon cataract, have in general been written for the exclusive purpose of recommending a particular operation; and it has been truly remarked, that too frequently the more favourable side alone of the subject has been exposed to public view."

If other writers have really been guilty of this partiality to their own practice, we may easily suppose that they have not been the less careful in shewing the unfavourable side of the contrary mode of operating; and, if Sir W. has selected the latter only from each party, it is easy to conceive what a hideous picture he may produce. After reading it, instead of *apologies* for altering his former practice, we should have expected his confession for having continued it so long.

Before we enter on the consideration of the proposed improvement, we must offer a few remarks on the introductory chapters. In the first, on the opinions of various authors, ancient and modern, concerning the nature and seat of cataract, much novelty could not be expected. We, however, read on, with a good deal of patience, through the mistaken notions of Celsus and Pliny, among the ancients. The moderns are too numerous for us to particularise: among them, Mr. Travers appears first and last; and it must be admitted, that Sir William has shewn no inconsiderable judgment in the choice of his adversary, whom he has assailed without mercy. At the end of all this, we have a slight mention of the black cataract. As we have frequently boasted how superior the English account of this disease is to the French, we examined with much attention how far our opinion of Mr. Stevenson's description of that form of cataract was confirmed by Sir William. But on this occasion we find no names quoted, no reference to the opinions of authors on the mode of distinguishing a black cataract from gutta serena; nor any hints by which Sir W. was led to perform the operation on Mr. W***, a brewer in Bath, who for twenty years had been universally supposed, by town and country practitioners, to labour under gutta serena. The universality of such an error might have induced the author to afford us some information of the means by which he was enabled so judiciously to form his opinion, and to determine on an operation so unexpectedly successful.

Two chapters follow, one on Depression, the other on Extraction. In the latter, the author's new plan is developed; and here Mr. Travers is again introduced. To do justice to all parties, we shall transcribe the passage.—

"I have already adverted to my practice of extracting the opaque lens, after first placing it with the needle in the anterior chamber, in cases where its nucleus is so firm as not to admit of division. This practice originated in having, while at Exeter, in 1810, experienced the beneficial results of extracting floating pieces of capsule; and also from having (in the cases of undivided nuclei, mentioned in my work on Diseases of the Eye, which occurred to me early in 1812,) witnessed the like favourable results, of extracting the nucleus of a solid lens, when, placed for absorption in the anterior chamber without division, it had excited a great degree of irritation by its mechanical friction against the iris. From these circumstances I was encouraged at once to extract a hard and solid cataract, without leaving it in the anterior chamber for solution and absorption, after previously ascertaining with the needle that, from its solidity, it would not admit of a division; and, accordingly, in the month of June of the latter year, I for the first time operated according to this new plan upon Mr. Israel, a gentleman of the Jewish persuasion, assisted by my nephew and assistant, Mr. Hockin, in the presence of the family-surgeon, Mr. Van Oven, a gentleman now practising in the city. The favourable termination of this operation, from which not the slightest inflammation resulted, determined me from that period to perform it in all similar cases. I accordingly, the same year, assisted by Mr. Hockin, operated on two other cases, by first placing the lens into the anterior chamber previously to opening the cornea. The latter step of the operation was, however, effected in a different manner from that which I have since adopted, and which will be hereafter described; having carried the knife across the anterior chamber, in the manner recommended by Mr. Wathen. In January, 1813, the Greenwich pensioners were placed under my care, by order of the directors, and I performed this operation on all the men whose cases required it. This, in common with many other operations on the eye which I am in the habit of performing, was witnessed by a great number of professional gentlemen, who were invited by the medical officers of that establishment, and by myself, to be present at their performance; and, during the entire year in which they were from time to time performed, I never remember to have operated once without the presence of some professional visitors. Dates and circumstances have been thus particularly noted by me, because I have sometimes had reason to complain of uncandid treatment. When a new operation in surgery, or the improvement of a well-known operation, is proposed, the public probably are not very much interested in determining to which individual the priority of invention should be allotted: the fact most important for them to settle is, under whose hands the operation has been most successfully employed. But the feelings of an individual, who is anxious for the advancement of his profession, will naturally lead him to submit, for the opinion of the public judgment, the claims which he believes himself to possess to their favourable consideration; and, by securing evidence of his own exertions for the public benefit, prevent others from wresting from him the degree of reputation to which his labours may have entitled him. For these reasons, a record of the operation, and the manner in which it was performed, was preserved by the surgeon of Greenwich Hospital, in the hospital books; and this fact is referred to in my Letter to the Directors, which was published, with other official documents, by order of the Board, and at the expence of the hospital. The necessity of this precaution soon became evident; for, in 1814, some months after the publication of the Greenwich Report, and two years after the operation had been systematically adopted in my practice, the new principle of placing the cataract in the anterior chamber previously to its extraction, was published by another practitioner, without any acknowledgment that I had previously adopted, and also adverted to it, in my Letter to the Directors of Greenwich Hospital. In a note, indeed, in Mr. Travers's paper, there is a case mentioned as having been operated upon in this manner twelve months before.

It may, perhaps, be said that Mr. Travers had not even heard of the operation in question having been performed by me; and also that, although his description of the operation did not appear until the latter end of 1814, yet that he nevertheless practised it previous to the period of my having exhibited it to a great number of individuals. I cannot, of course, take upon myself to decide the question as to the probability of his having heard of this operation, or otherwise; but I have two very strong reasons for believing he had never performed it himself previously to my operating on the Greenwich pensioners. The first is, that I have learned from a gentleman who witnessed all the operations at the London Eye Infirmary up to the latter end of 1812, that, whenever extraction was performed at that Institution, it was always done according to the usual method. The second is, that, in the case of Edward Turner, one of the Greenwich pensioners placed under my care, who had undergone thirteen operations in the London Eye Infirmary, (seven on one eye, and six on the other,) I found the cataract of one eye detached and floating behind the iris, where it still obscured four-fifths of the pupil. This was a species of case to which the operation in question was peculiarly applicable, as, without difficulty, I placed the floating cataract in the anterior chamber. Finding, however, that its nucleus admitted of free division, instead of extracting, I left it for solution and absorption. If Mr. Travers had been in the habit of performing this operation at that period, his humanity, I conceive, would have led him to have done so in this instance, rather than subject the patient to so many useless operations. I say useless, because I can positively affirm that *not one-third part* of the lens in either eye was absorbed when I operated upon him, which fact was witnessed by the medical officers of Greenwich Hospital, by Mr. Astley Cooper, and by Mr. Merley, late assistant at the London Eye Infirmary, who had seen Turner while a patient at that Institution.

"It has been submitted to me, by some of the numerous surgeons who have seen me perform this method of extraction, whether I am justified in having used the word 'novel' as applied to it in my Letter to the Directors of Greenwich Hospital, and whether it may not be said that St. Yves, in 1707, had performed a similar operation. I was perfectly aware of his having extracted a cataract, which had *accidentally fallen into the anterior chamber of the eye*, at the time I wrote that letter.

"The following is the passage where he describes the first case which had occurred to him, requiring the extraction of a cataract, which was lodged in the anterior chamber of the eye.—'The first was in the year 1707, in the presence of Mr. Mery, a member of the Royal Academy of Sciences. I performed it on a merchant of Sedan: he came to Paris on account of a shaking cataract which had passed through the hole of the pupil into the anterior chamber of the aqueous humour. The cataract, by pressing very much the iris, occasioned violent pains in his head, attended with the want of sleep for three months before. At that time I had never heard of the like operation; but, reflecting that I often opened the cornea, to discharge the matter of an abscess lodged behind it, I concluded I might safely do the same in regard of a solid body; and I performed the same operation.'* In other words, St. Yves, being thus called upon to remove a cataract, which, from its situation in the anterior chamber of the eye, was occasioning very severe sufferings to the patient, had no resource left but to remove it in the best manner he was able, and therefore extracted it by making an opening in the cornea (in a similar manner as he had been accustomed to discharge purulent matter which had formed in that cavity); not, however, with any view of restoring the patient's sight, but merely to relieve him from the acute pain produced by the pressure of this extraneous body against the iris.

"To have contended that the common operation of dividing the cornea for the purpose of extraction was novel, would be so obviously absurd, that those who may be inclined to dispute with me the novelty of the operation can never have pretended that the claims to originality were founded upon this basis. Yet this is

---

* See translation of St. Yves's work, by Dr. Stockton, p. 163.

the only part of the operation which resembles that of St. Yves; and, even here, my practice is more like that of Daviel than his: as St. Yves performed the section of the cornea by carrying the knife across the anterior chamber, which practice I abandoned from the unfavourable results I found attending it, and which method of accomplishing that step I condemn in the operation described by Mr. Travers, and just now alluded to.

"The operation performed by me is adapted particularly to those cases where the nucleus of a cataract is too hard to admit of immediate division. It consists of two stages:—the pupil of the eye being dilated by belladonna, the entire cataract is first placed, with the needle, in the anterior chamber of the eye; the cornea is next divided, and the extraction of the lens completed. What then, may I ask, has been borrowed of St. Yves? Surely not the whole of the operation just stated. It cannot be the removal of the opake lens into the anterior chamber of the eye; for that had been effected by accident three months before St. Yves saw the patient, and it would appear by the following passages in his work, taken from under the head, 'How to prevent the accidents which attend the operation of the cataract,' was a circumstance which had at first caused him considerable embarrassment. Nothing, then, could have been adopted from him, unless it be the application of belladonna, or the common incision of the cornea in the operation by extraction; which, if offered as any bar to my claim of originality, would be too absurd to require a reply."

By the quotation which follows, it is very certain that St. Yves never proposed this *double operation*, unless when accident or peculiar circumstances rendered it necessary. Whether two operations are preferable to one, we pretend not to determine; but, after what is quoted from St. Yves, we cannot help thinking that any dispute of priority of invention, Adams *v.* Travers, would be

——"rixatur de lana caprina,
Propugnat nugis armatus."

The work contains a great deal more, of which we might take notice, were there sufficient novelty to interest our readers. We shall only remark one among the objections against couching. We are told, that the absorbents have no power of taking up solid matter. How, then, do we find an opake capsule so often absorbed? how are bones absorbed? We shall be told that they are first dissolved. If, then, a hard cataract is broken from its attachments, why should not that also be dissolved? But to us solution appears not at all necessary to enable the absorbents to take up solid matter of any description. This is, however, entering on a new question; and we trust nothing that we have said will induce the reader to suppose we are urging a preference to any mode of operating. Couching has always appeared to us the most simple, but we readily admit that a cataract may be so hard as to render extraction the most desirable practice. We can even conceive cases in which the double operation may be preferred, but are not prepared to advise its general adoption.

We here finish our remarks on the "Practical Inquiry." The "Letter," not being addressed to professional men, may be thought unfit for our Journal. We admit that the directors of Greenwich Hospital are not likely to peruse our pages; or, if they do, to understand them. Appeals to the public on professional subjects have always a suspicious appearance. On this occasion, therefore, we are as little satisfied with the answers from the "Eye Infirmary." Most of all, we feel disappointed that the medical officers of an Institution, begun under such auspices, should be appointed from among the general practitioners in surgery, and in one instance, we are informed, from the practitioners in pharmacy. This is not the way by which a practical art is to be perfected. To do justice, however, to Sir William, he does not confine his remarks to extra-professional readers: in an Appendix, the Edinburgh Medical and Physical Journal comes in for its share; and Mr. Stevenson is still more roughly handled. The editors of the above respectable publication will doubtless defend themselves. If our valued correspondent, Mr. Stevenson, will honour us with his reply, with the same urbanity as he has hitherto shown, we shall gladly find room for his communication. As medical readers are the only competent arbiters in such controversies, so the only arena, in our opinion, is a Medical Journal.

We cannot, however, entirely dismiss this article without an inquiry particularly interesting to us, as connected with the subject of Infection or Contagion.—What, let us ask, is the *Egyptian ophthalmia*? and what is Sir William Adams's improved method of treatment? Respecting the first, whatever its cause, the effect, as far as we have been able to learn, is either high inflammation, or inflammation ending in ulceration, suppuration, or slough. The mode of treatment, we conceive, must be similar to the mode of treatment in other cases of inflammation; but, in the diseases of so delicate an organ, the necessity of very early and very bold remedies is, if possible, greater than in most other parts. The Egyptian ophthalmia, as it is called, having appeared principally among the soldiery, we might expect the treatment should be such as is found requisite in the treatment of inflammation in all other cases among men in the vigour of life, and kept continually in a preternatural state of animation. Such being our opinion of the disease, if Sir William has found out a readier way of reducing inflammation than was before in use, we cannot doubt that it will be generally adopted, and that he will find his reward in the credit he must obtain among his brother practitioners, and gradually with the public at large. But, if the claims he sets up depend on whether tartar emetic shall be given in nauseating doses, or so as to produce vomiting, we conceive that, after the free use of the lancet, as recommended by Dr. Jackson (see our Collectanea), tartar emetic may be given in doses of any description, or even omitted altogether.

Having touched on the subject of Egyptian ophthalmia, our readers may expect some remarks preparatory to our promised paper on Infection. Egypt has ever been known as the source of contagious diseases of all kinds. A country in which the atmosphere is rarely disturbed by hurricanes,—which is almost a stranger to rain,—which has been for so many years consigned to the most abject poverty, and its attendant miseries,—cannot fail to be continually a prey to that infectious atmosphere which, under different circumstances, induces the hospital or camp fever, or scurvy, or those local diseases which the most experienced writers describe as vicarious of such fevers. From the meteoric causes above mentioned, an epidemic ophthalmia, when once excited by the presence of such a tainted atmosphere, is likely to spread more universally than in any other quarter. Not only from the manner in which the atmosphere, by the slowness with which it moves, must be loaded with deleterious particles, but from the vast number of insects which multiply without the interruption of frost or rain, and which, perpetually fixing on the moistened parts of animals, may convey infectious particles from a diseased to a sound eye. Ophthalmia, it is true, has spread in England, though with less frequency than in Egypt. For this, various causes have been assigned by different writers, who have seen most of the subjects under the complaint. We are perfectly satisfied with the remarks made by Dr. Jackson on sore legs, eruptions on the hands and other parts; and our readers will recollect what we have lately said on certain appearances about the *pudenda* of very young virgins, of epidemic affections in the throat, and in other parts. We regret that these subjects have not been traced with that accuracy and minuteness to which their importance entitles them, and without which no pathological fact can ever be fairly ascertained. Dr. Jackson's remarks must, we conceive, eventually lead to this desirable end.

We shall here close a subject we have entered on with extreme reluctance. Without engaging in any personal disputes, the reader will perceive our opinion of the querulous language in which these works abound. If Sir William values himself so much for being the first in prescribing vomiting in severe ophthalmia, we shall not question his claims; though the late Mr. Saunders seems not to have overlooked it, by the following passage :—

"It may, therefore, be right, after the exhibition of cathartics, to employ the tartarized antimony in moderate doses, in order to enfeeble the pulse. If vomiting be excited by it, I see no cause of regret, as the straining of the eye in the act of vomiting is more than compensated by the weakness of the pulse which the state of nausea produces."—*Saunders's Treatise*, p. 27.

As to the other discovery—the double operation, we can see no advantage it possesses beyond the common mode of extraction; and the danger of inflammation would appear to us greatly increased from connecting it with the puncture in the posterior part of the orb. But time and experience must determine the question.

---

**[Having devoted so much of this Number to Ophthalmic subjects, we trust the following, from our correspondent at Hamburgh, will not be unacceptable.]**

*On the Operation of Keratonyxis, or the Cure of Cataract, by Means of the Needle passed through the Cornea and Pupil into the Opake Lens;—in England, denominated the Anterior Operation.* By W. Mensert, &c. Communicated by Dr. Von Embden, of Hamburgh.

The new method of operating for the cataract, by puncturing the cornea, has, for these many years past, attracted the notice of the Dutch oculists, and induced the skilful operator, M. Mensert, to communicate the result of his extensive practice in that branch.

In the first section, he treats of the operation of the cataract in general. After a view of the two methods, viz. extraction and depression, he introduces the new one of operating by puncturing the cornea. True and impartial are his observations on the zeal of other oculists in defending their respective methods, and on the success with which the famous De Wenzels, (father and son,) Beer, Marsinna, Arnemann, Demours, Forlenze, and others, have performed extraction; whilst, the no less celebrated Scarpa, and many earlier and later operators and ophthalmologists, have spoken in favour of depression. After the instruments invented from Daviel's time to our own, the author is led to conclude with Richerand, that the success of the operation does not depend so much on the mechanism of the instruments, and still less on the method employed, each of which may have its advantages according to circumstances, but chiefly on the skill of the operator, and the handiness with which he uses his instruments. Applying these remarks, likewise, to the indiscriminate praise which Buckhorn and Langenbeck bestow on the new method, he says, nobody will deny the success which De Wenzel and others have met with in extracting the cataract; nor will any one dispute the success of Scarpa, and others, in depression. It is the peculiar empyrico-practical and technical talent, according to which the merit and success of an operation, and the choice of the instruments are to be appreciated. Hence it is, that Richter, Beer, Mursinna, Arnemann, Schmidt, Himly, Langenbeck, and many others, in Germany; the De Wenzels (father and son), Dupuytren, &c. in France; Scarpa, Assalini, Quadri, and Monteggia, in Italy; have all of them most successfully operated in a peculiar way, and with instruments, either invented or altered by themselves: and, though the author himself is rather in favour of extraction, he thinks that every method may have its peculiar advantages; for which reason, he deems it highly necessary, that every oculist should be acquainted with them all, in order to chuse that method and instrument which may best suit him, and is best adapted to the circumstances.

In the second section, the author gives his ideas on the origin and improvement of that operation, called Keratonyxis; confessing, himself, that the observations of Gleize and Conradi, and, afterwards, the instructions of Richter and Reil have led his attention to that operation. The chief point on which the progress and future perfection of this method depends, is the solubility of the opake lens in the aqueous humour. To this solubility of the lens, and its absorption mentioned by former and later writers, the author dedicates a separate division, from which we extract the most essential points.

Ambrose Paré, as early as 1561, observed the solubility of the opake lens, and the disappearance of the remaining substance. Bartisch, in his 'ΟΦθαλμωδȢλεῖα, in 1583, observed the same. Guillemeau, Barbette, Antoine, Manget, and Daviel, have plainly hinted at it. Afterwards, Walther Curiensis, Rhaltus, and Molineux, paid particular attention to the absorption of the opake lens. The celebrated O. Acrell, by examining the eye, *post mortem*, cleared this point beyond all doubt. After this, Richter, Pott, Van Wy, Du Pui, Scarpa, Richerand, Beer, Fleury, and many others, though favouring different methods, have confirmed the absorption of the lens, after being depressed and broken down. This suggested to Wilburg the idea of changing the perpendicular situation of the lens into an horizontal one. The author, however, considers the power of absorption to be limited, and admits, from his own experience (made both in France and in Holland,) that the lens does sometimes remain undissolved after depression. Neither are his observations on the new method of operating for the cataract, though favourable on the whole, such as should lead him to give implicit credit to that power of absorption. That the solution and absorption of the lens and capsula depend on the peculiar nature of the cataract and its capsula, on the favourable disposition of the body, on the operation itself, and on the lens and capsula being properly separated and broken down, so that all the cohesion with small fibres and vessels, and thus the nutrition of the cataract, may be perfectly destroyed, and the whole washed away by the aqueous humour, which tends to soften and dilute the cataract, and to assist the absorbents in the performance of their office.

Having thus proved, that the absorption of the lens, the principal point on which the success of this new operation depends, has been known to former writers, the author examines, in the second division, how far this mode of operation is still to be considered as new. This method owes its origin principally to chance, some unsuccessful experiments, and, lastly, to the newly-discovered effect of the belladonna and hyosciamus, in dilating the pupil. If the defenders of depression had been acquainted with the effects of these two last remedies, there is no doubt but they would have operated by puncture. Avicenna, however, perforated the cornea with a depressing needle, with which he penetrated to the interior of the pupil. At the time of Albucas and Guy Van Chauliac, they pierced the cornea, passing through the pupil with a hollow needle, in order to remove the cataract; and, that the paracenthesis (puncture) of the cornea was in common practice at all times, may be seen from Sprengel's History of Physic, Hecker's Annals of Medicine, Haller's Collection of Theses, &c. Gleize's *Nouvelles Observations sur les Maladies de l'Œil*, Conradi, Richter, Beer, and others. The author, however, agrees, that the merit of having reduced this operation to a methodical form, and of giving it the Greek appellation of Keratonyxis, cannot be denied to Buckhorn.

The third section contains some proofs, that this method has been practised, and extolled also in France and England, and that too, about the period when Buckhorn made it known; his method is, however, said to be preferable to that of the French, to which that of Mr. Saunders's deserves to be preferred. Bannister was not unacquainted with the solubility of the opake lens. Pott knew its merits; but, nevertheless, the best oculists, as Saunders, Adams, and Stevenson, do decidedly prefer the absorptive process before extraction in the soft cataract. Mr. Saunders, and other English operators, know perfectly well the effects of belladonna and hyosciamus upon the iris, which, undoubtedly, not only helped towards the discovery of this new operation, but, according to our author, was the first occasion of it. Dr. M. wishing, however, the opake lens to be removed, not only with the least danger, but also in the safest and most certain manner, proposes first to dilate the pupil by exhibiting belladonna or hyosciamus, and then to depress the cataract through the sclerotica, which process he thinks in some cases preferable to puncturing the cornea, though the perforation of the former is more frequently attended with danger than that of the latter. He even thinks this method of depressing the cataract with a fine and sharp needle easier and safer, and the movement of the needle less confined than in puncturing the cornea. The English operators, he remarks, coincide in this idea, and he thinks much may be expected from it. This is followed by some extracts of letters, respecting the ophthalmoïatric of the English, in which honourable mention is made of some English oculists, as Adams, Stevenson, and Saunders; particularly of the latter, who is said to have first introduced the operation through the sclerotica, after the exhibition of belladonna, without depressing the lens.

*Observations respecting the Keratonyxis.*

The first time the author attempted Buckhorn's method, was in a man 59 years of age. The capsule was easily lacerated; the greater part of the opake lens could not, however, be broken down; he, therefore, pushed it along the narrow margin of the pupil, and then effected the depression through the cornea. After a month's time, the patient had pretty well recovered his sight. The author thinks this proceeding always indicated where a *cataracta dura* exists.

In a woman, 70 years of age, depression was performed. After four days, the pupil had assumed an oblong shape, and the cataract had resumed its former position. In thirty-eight days, the pupil continued perfectly covered, and no trace of absorption was observable. The depression through the cornea was now undertaken for the second time, and with success. In the first operation, the author had made use of Scarpa's needle; in the second, of that of Langenbeck. He thinks, however, that this second operation might have been spared, as absorption would have taken place without it.

*Cataractæ adhærentes capsulares.* In this case, neither hyosciamus nor belladonna had any effect; the operation was somewhat difficult; yet, it succeeded so far, that the patient might have left the hospital with his sight restored, had he not died of a catarrhous fever. On examination, *post mortem*, the lens was found much softened and diminished in its bulk, the capsula adhering to the iris had, for the greatest part, remained, and the vitreous substance was unhealthy and liquid. In cases like this, the author thinks the puncture to be contra-indicated, as the opake capsula will seldom, if ever, be dissolved. A woman, of 68, was also, though but slowly, restored to sight by puncture and depression. A child, of eight months old, underwent the operation twice, without absorption. A man, 83 years of age, was punctured in both eyes; both the lenses were, with difficulty, broken; and the left pupil contracted violently, whilst that eye was operated upon; inflammation followed, which proceeded to hypopion, the cornea became wrinkled, and the eye collapsed; but, the right eye, notwithstanding the prevailing sympathy, regained its vision. In a woman, affected at the same time with amaurosis, the operation of keratonyxis was undertaken at her particular desire; the breaking down and absorption, succeeded pretty well, but her eye-sight remained lost, as the operator had presaged. In a girl, 13 years of age, and another, not yet a twelvemonth old, the operation was successful. A case follows, in which the lens mounted again, and, an arthritic inflammation coming on, induced the adhesion of the pupil to the again-ascended cataract. Then follows another case of iritis, and the whole is concluded by two successful cases.

The author performed this new operation on twenty-eight eyes in the course of four years; of which, six cases were perfectly, and eight partially, successful. During the same period, he performed, on one-hundred and seventy-eight, the operation of extraction; of which, one-hundred and thirty-two succeeded perfectly, nine but partially, and thirty-seven not at all.

The work closes with a consideration of those cases in which the new operation deserves the preference. In general, extraction is considered the safest, and, for a well-practised hand, the best method; in certain cases only, this new method deserves to be preferred. The cases particularly applicable are cataracta congenita, fluida, and lactea, as also in deeply-seated eyes, and where the eye-lids cannot be opened at a sufficient distance. In very timid patients, who would not submit to a second operation, or in whom, on account of convulsive motions of the eye, extraction is not admissible; where the cornea is affected by maculæ or other diseases, in sickly or very aged persons, who cannot, with safety, submit to extraction, on account of the great restrictions, care, and quiet, which it requires; and, in such cases, where any particular predisposition prevails to inflammation, chronic blepharophthalmy; or, where an amaurotical state of the eye leads the operator to fear the consequences of extraction.

---

*An Essay on the Human Ear, its Anatomical Structure and incidental Complaints; intended not only for the Medical Profession, but also for the Use and Benefit of all Persons afflicted with Deafness, Diseases of the Ears, or those alarming Sensations of Noises in the Head. Including Remarks on the Causes and Increase of the Deaf and Dumb; with Cautions highly interesting to all Mothers and Families; by Attention to which, such a Calamity may be averted.* By W. Wright, Surgeon-Aurist, and Lecturer in Natural Philosophy and Chemistry, No. 7, College-green, Bristol.—8vo. pp. 123. Longman and Co. 1817.

Whilst transcribing this long title-page, we were attracted by an engraved Booby-countenance, which stands as a frontispiece. Hastily conceiving it to be a head of the author, we were surprised he should have suffered the artist to represent him so disadvantageously. On a closer examination, we found it the head of a poor unfortunate boy under examination of the *auditory passage of the ear*, by this lecturer in natural philosophy and chemistry. Without this figure, we could readily have formed an idea of the manner in which the finger and thumb of the left hand holds back the auricle, whilst the right introduces an instrument. "The work is intended not only for the benefit of the medical profession, but of all affected with diseases of the ear, and contains cautions highly interesting to mothers and families;" we shall content ourselves with transcribing a few passages, which, to us, are so obscure, that, measuring the abilities of others by our own, we are fearful no one but the author can find any advantage from his Essay. If, as Mr. W. pleads, his professional duties allow him but little time to correct his style, we recommend him, at least, to accept of the assistance of others. The following passage, being partly auricular and partly chemical, seems well suited to the author's acquirements.

"It is well understood, that the skin possesses absorbent and excretory vessels; the latter of which, we need only take into consideration. From chemical analysis, it has been discovered, that the nature of these excretions are nitrogenous, or truly alkaline in their properties: now, if we mix certain proportions of this gas with atmosphere air, a reduction in volume will be the result, as the oxygen contained in common air is absorbed, or, I may say, annihilated, in consequence of the mutual attraction; and, reasoning upon the fact, that atmosphere air is composed of solid bodies, expanded by caloric or heat into aerial vapour, my argument will not appear chimerical, in supposing that oxygen, which forms one of its constituent parts, should produce an absorbent action on these excretions; especially, when we daily see metals and every other substance decomposed by it, and, as the health varies, so the cerumen, by an alteration in its nature, may have less of that property which the oxygen of the atmosphere can neutralize, and accumulations of gross impurities are, therefore, deposited, occasioning stoppage in the ears.

"Sometimes the auditory passage is incrusted with a species of dry scurf, or scales, and on analysis these exudations appear, in some instances, to be dried fibrin, and, in others, albumin, frequently joined with ceruminous particles. These cases are very common, and are generally attended with noises in the ears, pains in the head, and often a tendency to constipation; from there being no visible cause, it is ascribed to nervous affections; the whole class of nervines are immediately resorted to, until, at length, the patient, tired out with medicine, sits down satisfied of his or her nervous debility, and hopeless of relief."

"Allowing that it is either albumin or fibrin, of which the exudations are chiefly composed, it will seem there must be a superabundance of these qualities in the blood; and, as it is well understood, that the whole class of nervines promote coagulation, it necessarily follows, that the evil must be increased very considerably by persisting in such a course.

"The late Mr. Saunders treated several of these cases, as he considered, successfully, upon the antiphlogistic plan, and, although the calomel, cathartics, and blisters, he used, might give relief for a time, yet I have reason to believe, these methods will never effect a radical cure; indeed, as the scientific, and, I may say, conscientious gentleman, Mr. Saunders, gave up his Dispensary for Diseases of the Ears, in 1814, (as appears by the Monthly Magazine for March in that year,) in consequence of his thinking no means existed for remedying the defects of that organ; and, an intimate friend of his assured me, that, out of 1200 cases of this species of deafness, six only were relieved. I feel myself warranted in saying, that I do not think the means he used, adapted to attain the desired end; and, from a conviction of their inutility, upon physical reasoning—a consideration of their chemical action—my own early experience—and the sufficient trial Mr. Saunders gave these methods, I shall never be brought to believe they can be usefully applied to relieve the extreme cases of this nature, frequently presented by the persons who are deaf and dumb, until men of science and character, after full and patient experiment and investigation, prove my opinions erroneous; for which I shall accept no evidence except their testimony, or that of *persons* who are *above* being *influenced* by any *consideration* than that of *truth!!!*"—Mind that, Reader.

*Observations relative to the Use of Belladonna, in painful Disorders of the Head and Face; illustrated by many Cases.* By JOHN BAILEY, Medical Practitioner, of Harwich.—London: Highly and Son. 8vo. pp. 72.

To invent a new remedy for a disease considered almost incurable, appears to us more meritorious than to discover the means of making two blades of corn grow where one only was accustomed to thrive. First, because there is still remaining space enough in the world to nourish a million times its number of inhabitants on the present mode of agriculture, and an increase of population always produces an improvement in the arts, and a larger supply of the means of cultivation. Secondly, because the inventor of an improvement in agriculture reaps the largest, and, for some time, the only, advantage in such an invention. But the discovery of a specific for a disease, is the procuring that which can no otherwise be procured, and the public for the most part reaps the whole advantage. The inventor is rarely the patient, and, if, as in the present instance, a regular practitioner, he cannot, according to the laws of the profession, make a secret of his discovery. Probably Jenner is the only man of this description who has ever been rewarded by the public. The work is dedicated to our former colleague, Dr. Shearman, to whose merits a due tribute is paid. A Preface follows, and also a short chapter, each containing good matter but delivered with more pomp than usually precedes such useful information as we find in the work. However, the last paragraph of the "Observations" must not be passed over. It affords an admirable lesson to the profession of what they are to expect in the practice of medicine, and how well they should feel themselves rewarded, if, without pecuniary aids, they enjoy those luxuries to which the author so feelingly alludes.

"It has been the lot of the author of the following sheets to toil for the last twenty years in a wide field; out of which, no path has led either to wealth or fame. To be useful has been his chief endeavour; and the only riches which he has gathered, or can hope to obtain, are self-gratulation and the luxury of doing good."

By an accidental circumstance, Mr. Bailey discovered, that the narcotic properties of belladonna affected, in a peculiar manner, the sensibility of the integuments of the face, and also of the lingual and optic nerves. That, contrary to the general report, the abdominal and pelvic viscera were little affected. We may, however, remark, not from personal knowledge, but from the recollection of well-ascertained cases in which the fruit was by mistake taken by children, that the effect on the bowels was very considerable. This is not mentioned by way of contradicting, or even instructing, Mr. Bailey, but as an apology for that apparent discrepancy between his and the popular opinion. In the work before us, the extract made of the leaves, according to the London Pharmacopœia, is recommended, and also a tincture made of two drachms of the dried leaves, and one *octarium* of proof spirit, steeped for twenty days. Of the extract, it is found adviseable to begin with three grains four times a-day, rather than with a smaller dose. This, we conceive a fact of no small importance, because, by an unnecessary caution, the constitution may be so gradually familiarized to a vegetable narcotic as to deprive it of much of its efficacy. But a case which has occurred since the perusal of this work, convinces us, that in this, as in many other remedies, the constitution of Londoners is more easily affected than those of the inhabitants of the country.

In considering the disease, the author is led to assign what he calls *Neuralgia facialis* to the decayed state of the *molares*. It is no objection with him, that the disease will sometimes continue after these teeth are removed, because it is well known, that a nervous sympathy excited may be continued after the exciting cause is removed. Hemicrania is considered as only another form of the disease. This opinion of the author is strengthened, by tracing a somewhat similar affection in a more distant part of the body.

"A stout and vigorous man, aged sixty, whose constitution had been hardened by exposure to many an angry winter's wind, and whose mind was rough and sturdy as his frame, became timid, effeminate, and irritable, from a long and debilitating attack of Neuralgia Cruralis. Every local and general remedy was employed, and pertinaciously and resolutely kept up; but with no diminution of uneasiness. Ease, however, one day came unexpectedly, whilst he was trying, by the aid of his elbow-chair, to raise himself upon his feet. He could bear the foot of the affected side upon the ground, and sustain the superincumbent weight of his body upon the limb, with as much freedom as at any former period of his life. In short, from having been confined for many months to his house, and tormented with shooting pains up the limb and along the side of the body every time his foot touched the ground, he became a sound, healthy, and active man. A month afterwards, a jagged urinary calculus, about the size of a large pea, was extracted from the urethra. The passage of this substance through the ureter into the bladder, explains the nature and cause of his sufferings."

The author attributes the temporary effects induced by mercury on tic doloureux, entirely to the excitement of a different action on the salivary glands, which are so immediately in the neighbourhood of the nerves affected. In all cases of neuralgia, therefore, at a distance from these glands, he conceives mercury has produced no advantage whatever.

Of the cases, which are twenty in number, we select the three first in order.

"Case I.—In the autumn of the year 1811, a married man, of an irritable habit and delicate form, was tormented with a most severe pain, which commenced its attack over the orbit of the left eye. It was not of the continued kind, but arose occasionally, and darted down the cheek with great fury. The molares in the upper-jaw were in a state of decay; but, as they were numerous, the patient did not choose to have them all removed. In the beginning of the month of November, an oblong blister was applied over the orbit of the affected part, and another behind the ear of the same side: he took, likewise, considerable doses of ether and opium. The state of his bowels was duly regulated, and a cautious attention was directed to the avoiding of those external causes which had induced the attack. Having persevered fruitlessly for a long time, he grew impatient, and wished to try something else. Six grains of extract of belladonna were ordered for him, to be divided into six pills; one of which was to be taken every six hours. In twenty hours he took four of the pills, and, obtaining perfect ease, he thought it unnecessary to employ the remainder.

*Observation*—During the residence of this person in the neighbourhood for two years afterwards, he had no return of his complaint. From the number and state of the molares, being almost all decayed, it is likely, that, by this time, he has been a sufferer in the same way more than once.

Case II.—A married healthy-looking woman, mother of three children, had, at various periods of her life, suffered considerably from the decayed state of all her teeth. She applied to me in the month of October, 1812, for a most severe pain on the right side of the face. She had been under the care of a medical gentleman, who had tried in vain the remedies usually employed on such occasions. The pain could clearly be traced to a diseased molaris, which she showed some anxiety to have removed; but, the recollection of the former case being fresh in my mind, I determined upon trying the effect of belladonna. At the time of her application, she expressed herself to be in great distress, and would have cheerfully submitted to the trial of any means, which might probably afford her the smallest relief. She took six grains of the extract in the course of twelve hours; a quantity much beyond what she was ordered to take. It produced its usual disagreeable sensations in the throat, but all her sufferings entirely ceased, and have not since recurred.

"The complete and perfect removal of pain in these two instances, determined me upon seeking for opportunities of putting the powers of belladonna, in this kind of ailment, to the test.—The following cases, selected from many others, will show to what degree of consideration it is entitled.

"CASE III.—Mrs. D. aged about thirty-five, had been a long time afflicted with severe pains on the right side of the face. It attacked her with the greatest violence whenever the upper and lower teeth came in contact, particularly in eating. The pain was of short duration, and produced upon the application of the most trifling external cause. The gentleman under whose care she had been previous to her attendance upon me, advised the removal of two of the molares, which she had consented to. Obtaining no relief from that operation, and tired of medicine, she lost her patience; belladonna, in the manner directed in the former cases, was ordered for her. Some relief followed; but, as her sight became much affected, she discontinued the pills. However, upon receiving an assurance that the imperfection in her sight was a temporary effect of the pills, she again resumed them, and ultimately lost all uneasy sensation."

The following is selected as a case of hemicrania.

"Mrs. T., a married woman, aged upwards of forty, has been several times afflicted with severe hemicrania. In July, 1815, she was again attacked, and applied for my assistance. A medical friend, then on a visit at my house, saw her in my absence, and ordered a blister, with the other usual means; but, desirous of witnessing the effects of belladonna, before any other medicine was tried, I directed that she might take two grains and a half of the extract in a pill at bed-time. On the following morning, having been visited by this gentleman, he reported her as quite free from pain, and that it was unnecessary to use the means which the day before he had recommended."

We select part of the twentieth case to show, that Mr. Bailey is not backward in relating an instance in which he has not been so completely successful; and, also, as a means of connecting some supplementary remarks.

A female patient had sufficiently recovered to wait on a family at Margate; from which place she returned greatly improved in health, strength, and appearance.

"Three weeks after her return to town, she came to me, with a recurrence of the pain; and, as it was inconvenient for me to see her often, I ordered her a box of belladonna pills, which she was to make use of at her own discretion. I learn that the tic occasionally visits her, and seizes upon the angle of the mouth as formerly, but that she puts it away by taking a pill or two whenever it prevents her eating. I feel disposed to believe, that, if this patient could fairly submit to the full operation of the remedy, it would annihilate the disease. The disagreeable consequences of large and repeated doses, offer an objection in her mind to a trial; particularly, as she can temporize with the disease, and make her situation comfortable, by comparison with the forlorn and wretched lot, which she at one time thought would always be her portion.

"In the foregoing cases, I have forborne to enter into a minute detail of every occurrence attending their rise and progress; and, also, of the effects that have not any reference to the action of the medicine upon both the disease and the constitution of the patient.

"To dwell upon the number of pulsations in a minute; the state of the tongue and skin; the quantity and quality of the ingesta; and the diurnal state of the excretions and secretions, would be to encumber the more useful parts of these observations with an uninteresting and tiresome prolixity of description; and to enlarge the bulk of this little volume, without adding any thing to its usefulness.

"The number of cases which I have selected out of many, have been few: they are, I trust, though much condensed, sufficiently clear, to show the striking influence that belladonna exercises over those chronical sympathetic irritations that particularly belong to the head and face, which harass and distress the sufferer to an almost interminable length, and which hitherto have shown an unyielding obstinacy to the power of every other kind of medicine."

We have transcribed so much of this short tract, that the reader may be apprized of the spirit of the whole. Of course, there are, intermixed with the cases, many useful and minute observations, for which the reader must refer to the work, and such will, doubtless, be the determination of every practitioner who has one of these tedious cases on hand.

---

*Medico-Chirurgical Transactions, published by the Medical and Chirurgical Society of London.* Vol. VIII. Part I.— Longman and Co. 1817.

*(Concluded from our last.)*

*Observations on the Morbid Structure of Bones, and an attempt at an Arrangement of their Diseases.* By John Howship, Esq. Member of the Royal College of Surgeons in London; and Corresponding Member of the Société Médicale d'Emulation, in Paris.

The abilities and industry of Mr. Howship are beyond all question; but we almost wish he had kept these observations longer, or that they had been submitted to the examination of less partial admirers than he may possess in the Society. Though we ought not to consider a gentleman who has been so long before the public, and distinguished himself so often, as young in practice or in writing, yet we strongly suspect, if he should live as long as every friend to science must hope, that he will be glad to revise this paper. The same may, perhaps, be said of the productions of most other medical men; and probably in this, as well as in forming our opinion of the subject in general, we may be accused of an unreasonable fastidiousness. We are ready to admit there are passages which we might have passed over in a more ordinary writer; but which we feel obliged to notice in the sequel of a paper which we have before had so much satisfaction in commending.

Mr. Howship frequently alludes to Mr. Hunter's writings and his lectures. It would have been very desirable if he had always quoted the precise passage of the first, and produced his authority for the second. Considering, too, that the lectures have never been published, and that "Mr. Hunter was much more intimately acquainted with the principles of diseased actions in bones than any author who has yet attended to the subject," it would have been particularly desirable if this paper had commenced with stating the doctrines of that great pathologist; after which, Mr. Howship's improvement would have been read with increased interest, as confirming or correcting what had been done by so celebrated a character.

Having said so much, we shall principally confine ourselves to a few extracts, leaving the reader to form his own judgment, assuring him, at the same time, that we have been careful not to draw them in a manner which would render them deficient in perspicuity by being insulated.

After remarking Dr. Russell's unfounded opinion that "the dissolution of the sequestra in cases of necrosis is doubtless very much accelerated by the solvent power of the purulent matter in which it is surrounded," Mr. Howship observes,

"Now, with regard to the mode in which the condition of bone is changed previous to being taken up by absorption, there has been much diversity of opinion. Mr. Hunter admitted he did not know how absorption was performed, but he believed that the absorbents of surrounding living parts were capable of elongating themselves, and of absorbing dead bone, which we see sometimes partially, sometimes wholly, removed. Mr. Cruikshanks says, 'it is possible that, previous to the absorption of a solid, the parts immediately to be absorbed may be broken down, mixed with, or even converted into, fluids.'* One of our most deservedly eminent teachers in medicine† considers the affinity of aggregation as exhibiting the nearest approach to that species of cohesion by which the particles of earth in bone are held together; and the experiments of Mr. Charles Hatchett, as well as those examinations I have myself instituted, prove that the particles of earth in bone certainly possess an animal

"* See Cruikshanks on the Absorbents, p. 111.

"† Dr. Hooper."

medium, a reticulated gelatinous matter; and, although it is only in the bones of young birds that I have hitherto been able distinctly to see the ultimate reticulated texture, the results of the operation by which I have been in the habit of removing the animal matter from the bones of various animals, demonstrate the equal distribution of that principle, as the constant basis of every ossific structure.

"My own opinion is, that, in every instance where bone is absorbed, the process is commenced by the agency of some power exerting itself in the blood circulated over the part, by which the state of the animal principle is changed, and the particles of earth let loose, so as to be ready for removal by absorption; and, although we yet know very little upon the subject, I think I shall be able to demonstrate that the interstitial absorption of bone takes place by the different modifications of action of the same vessels, and the same membranous sheaths. There is the slow absorption incident to growth and health, and that which occurs in connexion with healthy inflammation of bone; besides the absorption that takes place during the existence of venereal complaints, and the use of mercury; and many others."

We cannot wonder if Mr. Hunter acknowledged his ignorance how absorption is performed, or *how* any other action is performed in a living body. All that a true philosopher undertakes is to trace the actions and the organs by which they appear to be performed. Finding that solid matter disappeared by the actions of the economy, and well knowing that certain vessels possess the property of absorption, he had no scruple in imputing the removal of such solid matter to the absorbents; and, when we consider what we know of matter, why should we make this great distinction between its solid and fluid form? Where, let us ask, is the necessity of Mr. Cruikshank's "breaking down and dissolving previous to absorption;" or to what does "this affinity of aggregation" lead? or who can question that bone contains animal matter? or why should the previous process of letting the particles of earth loose be required for absorption?

After a few other remarks, the author commences his Arrangement of Diseases in Bones.

"I. *Alteration of external figure, not arising from general swelling, but most commonly from a deposit of newly-formed ossific matter, upon the surface of the bone.*

"Including all nodous formations, and those accumulations of ossific matter constituting exostoses. These affections are, in general, the result of irritation or excitement of the circulation in the periosteum, in consequence of which, the texture of the membrane itself undergoes certain progressive changes, while the capillary arteries deposit the ossific matter, the quantity and appearance of which will be regulated by the peculiar nature of the excitement in which it has originated; occasionally, the appearances of exostosis are the consequence of the external part of the affected bone being separated, and raised above the general surface.

"The appearances produced by the formation of new joints, and those resulting from the ossification taking place in the periosteum subsequent to necrosis, as well as the irregularities of surface consequent to the union of fractured bone, will be included under this division.

"II. *Enlargement, from swelling of the original substance of the bone.*

"Under this head will rank the various appearances produced by spina ventosa; an affection in which the natural secretions, for the most part, form the contents of the tumour.

"The apparent readiness with which the bone gives way upon these occasions is partly explained by the circumstance of the excitement pervading the membranes lining the canals that are within the solid substance of the bone, as well as the membranous expansions contained in the medullary cavity; in consequence of which the pressure by which the bone is expanded operates in the most diffused manner, for, while the general mass of contents within is keeping up a degree of pressure outwards, the parietes of the bone are still further induced to unfold themselves, by the influence of the same principle being extended throughout the whole of the innumerable canals that pervade the more solid parts of the ossific structure.

"These affections appear to be produced by a peculiar modification of scrofulous action."

To us the whole of the above section is very obcure; nor are we at all better informed by the reference to "a peculiar modification of scrofulous action."

Consistently with the plan we proposed to ourselves, our extracts shall be more copious than our remarks. Having extracted the commencement of Mr. Howship's "Arrangement of the Diseases of Bones," we shall now offer the introductory part of his "Observations on the Diseases" of those parts.

"DIVISION I.—*On the Alterations of external Figure, consequent either to partial Swelling, or to a Deposit of newly-formed Ossific Matter, upon the Surface of the Bone.*

"In the consideration of the affections of bone included under this head, the appearances to be enumerated are exceedingly various, both as regards the causes, and the changes induced. Some result from a specific disease, dependent apparently upon the existence of the venereal poison in the system; others appear to be the purely local consequence of external violence, without indicating any tendency in the constitution to take up unhealthy action; although we occasionally see instances of a contrary description, where the slightest accidental cause, producing a disturbed circulation in the periosteum, gives rise to some affection of that membrane connected with extensive secretion of ossific matter, and eventually fatal disease.

"Under this head, I propose also to examine the appearances and structure of ossific matter, deposited, not as the consequence of disease, but as the natural means for repairing injuries of various kinds. This association may seem objectionable, as it connects the appearances of disease with the consequences of health and strength; but the various actions here alluded to must be allowed to possess certain features of resemblance to each other, which has determined me to include them all under the same general head.

"*On partial swelling of the external surface of bone.*

"Mr. Hunter says, in his Lectures, 'In some cases the bones themselves are swelled, and the parts evidently pushed out: how this should be is very difficult to conceive.' Now, the appearance here described is exactly that which occurs in many cases that are considered exostoses. The principle upon which the change in the figure of the bone takes place, I have endeavoured already to explain; and, as to the degree of alteration and extent of the affection, these will be as various as the varying circumstances which regulate the extension of the sphere of irritation in the vascular membranous expansions connected with the periosteum, and passing thence into the solid structure of the bone."

After what we said concerning partial quotations, we may be allowed to wish that the transcript from Mr. Hunter had been longer. This is the only remark we shall make.

*An Inquiry into the Origin and Nature of the Yellow Fever, as it has lately appeared in the West Indies; with Official Documents relating to this Subject.* By William Fergusson, M.D. Inspector of Hospitals, and principal Medical Officer in the Leeward and Windward Islands.

"As an introduction to the subject which I propose to discuss in the present communication, I shall beg leave to premise the following queries, which were put by the Army Medical Board, in consequence of the great sickness and considerable mortality that prevailed on board some ships, conveying black recruits from Goree to the West Indies.

"*Barbadoes; October*, 1816.

"Queries relative to the Regalia Transport, which sailed with black recruits from the coast of Africa, for the West Indies, in 1815.

"1. *Was the ship good, and her crew healthy, before the blacks were embarked?*

"The ship was good and her crew healthy, until she took on board a large quantity of green wood, a very short time before the blacks were embarked.

"2. *What was the state of the black recruits? Who inspected them previously to embarkation? and from whence did they come?*

"Many were embarked sick from hospital, with ulcers, fluxes, &c. It is supposed they were inspected by the surgeon who had been appointed to accompany them, but did not, on account of sickness. No account can be obtained of the tribes or nations to which they belonged, previous to their capture at sea, or their being brought to Sierra Leone.

"3. *Do you conceive that any thing in the ship, or in the state of her cargo, could have produced a very destructive fever on her passage to the West Indies?*

"A quantity of green wood, recently laid in on the coast of Africa, and foul ballasting that had not been changed for years, I conceive perfectly adequate to the production of the most destructive fever, under various modifications of leakage in any ship navigating the tropical seas.

"4. *Inform me, as far as you can yourself learn, of the treatment of the black recruits, before and after their embarkation, their diet, exercise, &c.?*

"They had no surgeon to attend them in any of their own transports, and could not possibly be properly attended by the surgeon of the convoy ship, however ardently active might have been his disposition so to do; nor was it possible to furnish the transports with medicines and dressings as they might be wanted from that ship, supposing her to be furnished with supplies for such a service, which she could not possibly be at Sierra Leone, where the want of medical stores was assigned as a reason for sending sick from the hospitals to the ships. Their diet was not suitable, either in the provision laid in, or distribution afterwards, as was proved to the Military Board that sat at Barbadoes, to inquire into the causes of the sickness and mortality on the passage.

"5. *The description of these recruits, whether young or old, and their previous habits and occupations, in as far as you can learn them?*

"They were mostly very young. Their description that of uncivilized Africans. Some of them of a very barbarous description.

"6. *Inform me of the number sick on arrival at Barbadoes, and describe particularly the disease under which they laboured, with the different modes of treatment which were pursued?*

"114 sick, labouring principally under dysentery, ulcers, and dropsy, or cachectic leucophlegmasia. The treatment of the first was conducted by mercurials, ipecacuanha, lime juice, and the mucilages; of the second by stimulating antiseptic dressings, combined with a generous antiscorbutic diet; and the third by deobstruents, tonics, stimulants, diuretics, &c.

" 7. *I request to know the total number sick on the passage, with the number of deaths, and the number sent sick on shore?*

" The convoy ship did not come into Barbadoes, therefore no communication could be held with the surgeon to ascertain the number of sick on the passage. The number of deaths was 52; the number sent sick on shore to hospitals 114.

" 8. *I request to know the strength of the detachment when embarked at Sierra Leone; the strength when landed at Barbadoes; and the number sick on arrival at Barbadoes?*

" The number embarked was 793; the number landed 741.

" 9. *I beg to be informed of the result in the number landed at Barbadoes, and the present state of the detachment?*

*(Signed)* 'J. M'GRIGOR, Director-General.'

" The number that perished in hospital in the course of two months, from 24th of August to 24th of October, was 70; viz. 53 of fluxes, 5 of dropsy, 9 of pulmonic complaints, 1 of ulcer, and 2 of fever.

" The number treated 288. There was no case of elephantiasis or unmixed scurvy seen amongst the recruits landed at Barbadoes.

(Signed) W. FERGUSSON, Inspector of Hospitals.

" In replying to the foregoing queries, I have confined myself entirely to the points which I was directed to answer; but, as the circumstances of health under which the crew and the inmates of the Regalia Transport arrived at Barbadoes, present an interesting field of investigation in regard to the infectious nature of tropical fever and dysentery, I have considered it my duty to throw every light in my power on this long disputed and yet undecided question."

We have so long made up our mind concerning the non-contagious property of a fever which exists only at certain seasons, and in certain latitudes, that we shall dismiss that part of the question, especially as Dr. Fergusson agrees with us in that opinion.

" As the question (says our author) will next naturally arise, how such a fever as that which destroyed so many of the crew of the Regalia, and attacked almost every one that came on board of her to supply the place of those that had perished, could spread so unerringly and prove so destructive without being infectious, I shall enter into it more at length.

" The quantity of green wood laid in at Sierra Leone on board the Regalia for fuel, must have been very considerable; for, after she had been several weeks in the West Indies, there were still as many tons of it left as, in the master's opinion, would serve for a voyage to Europe. The ballast, too, had never been changed or sifted from the time she left England, nor for any discoverable time before. It was what is called single ballast, small stones, with a considerable mixture of mud and other impurities; and when I examined it on board the Regalia, it had been much fouled by leakage from the water-casks. The ship, in respect to leakage, was far from being a dry ship,* and from that circumstance might, with better ballast (of iron or large stones), have proved a very healthy one; but the absorption of sea water amongst foul ballast and green wood, could scarcely fail to prove unwholesome. In other respects the Regalia, in all her apartments of cabin, steerage, and betwixt decks, was uncommon lofty and well aired, and, so far from being crowded, she had about double the tonnage for the complement of negroes she brought over that is commonly allowed for troops. She was excellently found in every species of provisions and stores, and her discipline and cleanliness were unobjectionable. In short, there was nothing in her nor about her that could either generate or permit the retention, if introduced, of the matter of typhus fever. The cause of disease was, therefore, I am clearly of opinion, to be ascribed to the green wood laid in at Sierra Leone, operating along with the foul ballast to furnish, when impregnated with the gases arising from putrid sea water, morbific miasmata, similar to those that on land arise from marshes when exposed to the influence of the higher degrees of atmospherical heat. Why this morbific power operated differently on the blacks and the whites, may be explained from the fact that the African is very rarely amenable to those influences that affect white men with intermittent, remittent, or yellow fevers. If they operated at all, therefore, on them, they must have produced some other disease; but I see no reason to attribute the dysentery of the blacks, from which so many perished,† to other causes than those that have been proved to exist, viz. the sending numbers from the hospitals of Sierra Leone to the ships ill with that disease, the want of proper remedies and medical attendance during the voyage, and the highly improper and dangerous change of diet, from one wholly vegetable to the ordinary ship's rations, which, on board the Regalia particularly, were served to those poor creatures during the voyage.‡ They were received into our hospitals without any extraordinary precaution, fear, or scruple: for, though the medical schools of Europe have raved for centuries about the contagion of dysentery, of which, as their professors seldom served in armies, or lived amongst organized bodies of men, they could know very little: every regimental

"* She had 32 inches of water in the well at the time I sounded her, and, according to the master's calculation, she made three-quarters of an inch every hour."

"† One hundred and eleven fluxes were received into hospital, independently of those that died on the passage, 53 of whom (very nearly one half) perished.

"‡ Vide Report to the commander of the forces, founded on the evidence produced before the Medical Board of Officers that sat at Barbadoes; a copy of which was sent to the Medical Board, dated 28th September, 1815."

surgeon who has served a campaign, or surgeon of a militia regiment who has been in an autumnal encampment on any of the downs of England, knows well that, however practicable it may be, through an undue accumulation of sick, and neglect of cleanliness and ventilation, to propagate a typhoid contagion under the leading form and features of dysentery, ulcer, pneumonia, &c., dysentery itself, under all ordinary circumstances of accommodation, is no more an infectious disease than hæmorrhoids or catarrh."

We shall now add the first part of the "Appendix."

"Extract of Inspection Report to the Commander of the Forces, on the State of the Regalia Transport on the 26th of September, 1815, then appointed to carry home Invalids to England.

"1. That the crew on the coast of Africa was healthy till the blacks were sent on board.

"2. That, about the same time that the blacks embarked, a quantity of green wood was laid in for fuel.

"3. That soon after the fever broke out, and several were taken ill, and two died the first day after sailing.

"4. That the crew continued to fall ill one after another on the passage, until all except one boy had suffered attacks of fever, and five out of twenty-one had died before arriving at Barbadoes.

"5. That the captain's wife sickened and died after making the harbour where the ship remained four days; and that the captain immediately after sickened and died, on the passage to the Saints.

"6. That the ship remained two days at the Saints, after which she sailed for Antigua, where she remained three days before returning to Barbadoes, during which time a mate that had been shipped at Barbadoes, from another of the African ships reported to be healthy, sickened and died. Also a boy that had been taken at Barbadoes from the Lord Eldon, then a perfectly healthy transport, fell ill and was sent to the hospital; and the apprentice to the ship, the only individual that had hitherto escaped, for the first time took the fever.

"7. That, on her return to Barbadoes, she shipped a new mate from a healthy Newfoundland ship, who, on the ninth morning of his being on board, was found by me in a state of fever, and sent to the hospital.

"8. That, during the passage from Sierra Leone, and the short voyages through the islands, she has been under a constant course of fumigations by fires and otherwise; that she has now been fresh painted, and is at present, and, according to every evidence that could be collected, has been, in as clean a state as possible.

"9. That all who died were affected with vomitings, and bleedings from the mouth, nose, and other places.

"10. That the Regalia shipped three fresh men about the time the crew commenced the work of clearing her hold, one of whom took the fever, and was sent to the hospital at Barbadoes; and another died at Guadaloupe of the yellow fever on her passage home."

Respecting the origin of the fever, we are by no means satisfied. The crew was healthy till the arrival of the black recruits, who were many of them under the diseases arising from confinement, bad diet, and other privations. Yet the crew had been exposed to this faulty ballast for its whole voyage, and to the green wood some time, (we are not told how long,) before the arrival of the recruits. As, therefore, the condition of the recruits was sufficient to induce the ship-fever in any climate, we can see no reason for assuming any other and more uncertain cause.

We perfectly agree with Dr. Fergusson, that men unaccustomed to the service by sea or land are but little acquainted with dysentery in its severe forms; but we are not less satisfied than Dr. Blane, that a dysenteric subject introduced among the sick on board a ship, at a particular season, will sometimes change the form of disease, and that dysentery will supersede another prevailing complaint.

*Rupture of the Stomach, and Escape of its Contents into the Cavity of the Abdomen.* By JOHN CRAMPTON, M.D. King's Professor of Materia Medica, and Assistant-Physician to Stephen's Hospital, Dublin. *With additional Observations*, by BENJAMIN TRAVERS, Esq. F.R.S. Surgeon to St. Thomas's Hospital, and Vice-President of the Society.

This case, which should rather have been entituled, Ulceration of the Stomach, resembles one which will be found in our 12th vol. page 165. The only thing worth remarking, in the additional observations, is an opinion that the continuation of the pain, after ulceration has taken place, arises from the irritation of the peritonæum and peritoneal coat of the intestines, by the fluid falling on them as it escapes from the stomach. In some instances the adhesion of the peritoneal coat of the stomach to a neighbouring viscus, preserves that cavity at the part ulcerated, and, by degrees, a cicatrix is formed. Where this is not the case, the continued pain and sudden fatality are imputed to the escape of the contents of the stomach.

We cannot, however, dismiss the paper without a caution we have often wished for an opportunity to offer. The violent pain from inflammation of the stomach has usually the denomination of spasm or gout: in either case hot carminatives are administered, and, because the pain by degrees ceases spontaneously, the same remedy is repeated at every recurrence, till at length a higher degree of inflammation occurs, ending in ulceration and death. Patients subject to these violent pains should be taught to watch the first symptoms of a returning paroxysm, and to apply instantly for the lancet.

*Cases of Fungus Hæmatodes, with Observations*, by GEORGE LANGSTAFF, Esq. *and an Appendix, containing two Cases of analogous Affections*. By WILLIAM LAWRENCE, Esq. F.R.S. &c. &c.

This paper contains a number of most ingenious remarks and very valuable hints. To a common observer it seems scarcely credible that a disease of such frequent occurrence should have been without a distinguishing name, and imperfectly described, until Mr. Hey called the attention of the faculty to it, gave it an appropriate name, and described it with an accuracy that we cannot find has been since improved in any material point.

Such are the contents of the first part of the eighth volume, which promises to improve our art much more than if the Society had all the patronage which they were once ambitious to acquire.

---

*Edinburgh Medical and Surgical Journal*, No. LIII. January, 1818.

Art. I.—*An Attempt to develope the Fundamental Principles which should guide the Legislature in regulating the Profession of Physic.*

THIS attempt is laudable, but, in our opinion, bolder than the author is aware of. There are certain *fundamental principles* which should pervade all transactions; and, when these are established, laws will be unnecessary. Where they are not, laws, we fear, will be insufficient. In commercial concerns, custom is the law. In medical concerns, custom has been long at variance with law; and now, law is required to sanction custom. But that custom is so perpetually broken in upon, and by those whom the law may wish to protect, and who would fancy themselves injured by the interference of law, that it would be extremely difficult to know how to proceed.

The very profession of an apothecary, as now practised, is by some said to be illegal: his proper occupation being only in his *theca* or shop. Others, on the contrary, urge, that the separation of pharmacy from practice is dangerous and absurd. All are forced to agree, that, in a free country, every one should be indulged in his own choice to whom he will commit his health.

There is a spirit of aristocracy which governs us all. The professions are courted by those who think themselves above trade, and some of these are allied to wealthy and even noble families. The rank in which they mix, and the emoluments they receive, place them in an elevated point of view. They seem to ennoble the profession, and all who cannot assume a somewhat similar rank fancy themselves degraded, whilst others fancy they degrade the profession. These, and a thousand other little passions, are the sources of disquiet to the professors, and we know of no remedy for them. Whoever engages in medicine should make up his mind to suffer mortification, wounded feelings, and to undergo labour. In the midst of all this, is he worse off than others who are constantly hazarding their property, and unsuccessfully labouring for an uncertain maintenance? We shall hereafter take up the subject more at large; at present, it will be enough to transcribe the appendix to this paper.

"In a matter so vitally important to the whole community, as that which is treated of in the preceding pages, and involving such various and conflicting interests, no representations should be admitted on mere individual authority, or which have not their truth established by incontestible evidence.

"Nó means seem so suitable or powerful for eliciting the necessary information from the several sources capable of affording it, or for establishing the great leading facts, on which general conclusions to be just must be founded, as the appointment of a committee of the House of Commons, to inquire into the state of the medical profession, and to point out what amendments appear to be required therein.

"Should the legislature in their wisdom think fit to appoint such a committee, its labours would be materially facilitated, and its scrutiny rendered more direct and effectual, if a comprehensive sketch were prepared, pointing out the more important objects of inquiry, and specifying the more essential data on which the committee should rest their conclusions.

"In the following pages, such a sketch is attempted; and it is presumed, that, if all the facts and circumstances referred to therein were duly ascertained, sufficient materials would be accumulated to enable the legislature to judge with much certainty and precision respecting the natural tendencies of the profession, the mutual dependence and connexion of its several departments with each other, and the relationship which the whole bears to the general community.

"The following documents are important, and would be readily obtained, when called for by authority.

"The statutes of the universities, as far as they regard the courses of medical instruction, and the inauguration of physicians.

"The courses of medical studies provided by the universities, the qualifications insisted on as requisite for obtaining degrees, and the nature of the proofs by which these qualifications are ascertained.

"The charters of the several Colleges of Physicians, their by-laws,—particulars of establishment,—extent and general appropriation of funds,—amount of fees levied from each member and licentiate,—and the extent to which the penal and restrictive powers conferred by the several charters are exercised.

"The charters of the several Colleges of Surgeons, their by-laws, courses of education, qualifications necessary for becoming members and licentiates,—state of funds,—appropriation thereof,—establishment,—fees levied from members and licentiates.

"The apothecaries' charters, or statutes of incorporation,—their by-laws,—courses of education,—state of funds,—appropriation thereof,—establishments, and fees of initiation.

"The foregoing documents and returns would be readily furnished, on suitable application being made to the proper officers.

"The following are objects of investigation by means of statistical inquiries.

"The actual number of medical practitioners, both regular and irregular, throughout the united kingdom, taken by counties or districts, with the peculiar distinctions and qualifications, of each practitioner, authenticated by solemn declaration of the individual, and verified upon oath, if required.

"If possible, it would be most desirable to obtain some return of the physicians, and other regularly-educated medical men, who have retired from the profession, from the impracticability of gaining a livelihood therein; or who, having qualified themselves for practice, have been deterred from entering on it, by contemplating the actual state of the profession; also of those who, still adhering to the profession, find their utmost endeavours rendered ineffectual, their prospects blighted, and all hopes of reasonable emolument cut off, by the irresistible competition of the general practitioners, and the obstacles which oppose their engaging in the line of general practice themselves.

"A return of this kind must necessarily be very defective; but, however imperfect, it would be one of high interest, and afford much valuable information.

"In order to ascertain the real state of medical practice, and the practicability of preserving and enforcing those distinctions in the profession which our present system of medical polity enjoins, and our several chartered bodies endeavour so strenuously to uphold, the following inquiries should be proposed to those individuals of the different departments, who might be examined before the committee.

"To the Physicians.

"How far they are required to officiate as surgeons, in drawing blood, prescribing for surgical ailments, &c.?

"What advantage or inconvenience is experienced by the physician from his not exercising the art of surgery, or at least performing its minor operations?

"To the Surgeons.

"How far they are engaged in the practice of physic? Whether they could possibly pursue the exercise of their proper profession without practising physic? What advantages accrue from surgeons dispensing also?

"To the Apothecaries.

"How far they practise physic and surgery? Whether the practice of dispensing does not impose on them an inevitable necessity of prescribing also? In short, whether they are not, to all intents and purposes, general practitioners, differing from the surgeon-apothecaries only by the inferiority of their original qualifications? Whether any just grounds of complaint could be substantiated, if the whole class were prospectively to merge in the class of surgeon-apothecaries, forming thereby one comprehensive body of general practitioners?

"As a fact illustrative of a peculiar connexion, subsisting between pharmacy and the higher departments, which selfish cupidity has given rise to, it should not be concealed, that there are both surgeons and physicians who make a practice of sending their prescriptions to particular druggists, and of participating in the profits. The writer of this article has some reason to believe, that the practice prevails but too generally. He has himself received a circular letter from a druggist entering on business, and which is now lying before him, in which it is stated, that 'twenty per cent. was allowed on physicians' prescriptions.'—Such a fact should be unequivocally ascertained. Should it prove true, it may be asked, in what do such surgeons and physicians differ from general practitioners, except in being less honest, and less respectable?"

It is enough to read the above, in order to prove the impossibility of the legislature protecting the public against such abuses. We will venture to say, that no man should become a general practitioner, as the modesty of some would call him, without being as well qualified as he can be made for every department. How then can this be accomplished? —By every shop being closed and every general practitioner receiving fees. Already the education is only defective from that want of leisure, which the care of the shop prevents from being progressive. No man in his senses supposes, that a practical art is learned at universities, and wherein else does the education of the general practitioner differ from that of the surgeon or the physician? But the latter two have more leisure for improving their practice by observation. If this is any advantage, ought not every one to possess it who undertakes the charge of the health of others? This is only beginning to show the difficulties in which the question is involved; hereafter, we may undertake to unravel some of them.

Art. II.—*Observations on the Directions given by various Writers on the Practice of Midwifery, for Turning the Child; with an Account of an improved Method of performing that Operation.* By JOHN BREEN, M.D. Licentiate of the King and Queen's College of Physicians in Ireland, late Assistant to the Lying-in Hospital, Dublin.

The proposed improvement by Dr. Breen, is, we doubt not, very judicious; and, perhaps, has not been so generally noticed as it deserves, though it is certainly not overlooked, and, we suspect, oftener than he is aware of, makes a part of the common operation of turning. It consists in searching rather for the knee than the feet. We conceive, when the knee is discovered, the business is in a good train, as it will not be very difficult to bring down the feet, and this often in the very act of bringing down the knee, as the leg will soon offer the most convenient grasp. At all events, we conceive, Dr. Breen's remark worthy the attention of the practitioner, and that the teacher should, with his machine, make this a part of the learner's exercises.

Art. III.—*Account of the Effects of the Piper Cubeba in Curing Gonorrhœa;* by JOHN CRAWFURD, Esq. Surgeon, Honourable East-India Company's Service, Bengal.

This remedy, it appears, has been attended with wonderful success in the Island of Java.

"The pepper, well pounded, is exhibited in a little water, five or six times a day, in the quantity of a dessert-spoonful, or about three drachms. It is unnecessary to remark, that abstinence from wine and all heating aliment is proper. The ardor urinæ ceases, the discharge grows ropy, commonly in forty-eight hours, and frequently in less, and the disease ceases altogether soon after. These, of course, are the most successful effects of the medicine. In some cases, the cure is slower; in a few, it has been said to produce swelled testicle; and, in a still smaller number, it has been found altogether ineffectual. The sensible effects of the medicine are exceedingly mild. It occasions, though not always, a slight purging; it imparts to the urine its own peculiar odour, and it promotes its quantity. Now and then it occasions a flushing of the face, and a burning heat in the palms of the hands and soles of the feet."

"With respect to the cubeb itself, it is the fruit of a pepper-vine, the produce of the island of Java only, for it is not known even in any of the rest of the oriental islands. In the Javanese language, it is called *cumucus*. By the Javanese, it is used in some diseases of children, but never in gonorrhœa. In Bengal, into which it is imported from Java, it is used in medicine; and, it would appear, from what has been already stated, in gonorrhœa; but, whether generally in this last, I cannot say. The European practitioners of that country are, at all events, ignorant of its use in the disorder. I have been told, but cannot vouch for the accuracy of any information, that the cubeb is imported into Europe, and given to public singers at theatres to clear the voice."

The treatment of gonorrhœa, when chronic, must often be empirical. Our Monthly Report will contain the account of an injection we have often found efficacious, though we do not recollect to have seen it prescribed in print.

Art. IV.—*On the Effects of Nitre,* [*taken in large quantity into the stomach.*] By JOHN BUTTER, F.L.S. Member of the Royal College of Surgeons, London; Membre de la Société d'Emulation de Paris; and Surgeon to the South Devon Militia.

Two ounces were taken on an empty stomach. Vomiting was induced immediately; first of the solution, then of blood. Water and solutions of mucilage were the principal remedies. From eight to twelve o'clock the patient drank and vomited about two gallons.

Seven days after the nitre was swallowed, though most of the symptoms had subsided, blood continued to be passed by stool; and, for some time after, spasmodic affections of the muscles occurred, similar to chorea. The patient was two months gone with child, but no abortion followed. We conceive, she owed much of her recovery to the vomiting which followed so soon after taking the nitre.

Some useful remarks follow, on the danger of applying nitre externally,—a practice, it seems, very common among farriers, from its supposed antiseptic qualities. But, from the observations of Orfila and Brodie, confirmed by the author, the most frequent consequence is to induce mortification.

Art. V.—*On the Anti-variolous Power of Vaccination.* By G. COLVILLE, Surgeon, Ayton.

Mr. Colville insists much on the appearance of the cicatrix to ascertain a complete vaccination. We have, however, found small-pox after a well-ascertained vaccine cicatrix, and that a subject, in whose arm the mark has disappeared, was still able to resist variola. The disappearance of the cicatrix, though spoken of by a French writer as frequent, is, we believe, uncommon in England; and that some such cases were in subjects who had resisted the vaccine, and afterwards the variolous poison.

